# P-1108. Influenza Vaccination Uptake Among Healthcare Workers: Barriers, Facilitators, and Innovative Strategies

**DOI:** 10.1093/ofid/ofaf695.1303

**Published:** 2026-01-11

**Authors:** Jomel Raju, Leena James, Maria Tom

**Affiliations:** St. Joseph's College of Pharmacy, Cherthala, Pala, Kerala, India; St. Joseph's College of Pharmacy, Elampally, Kerala, India; St. Joseph's College of Pharmacy, Cherthala, Pala, Kerala, India

## Abstract

**Background:**

Despite being a vital preventive measure, seasonal influenza vaccination among healthcare workers (HCWs) remains suboptimal in many Indian hospitals. Understanding barriers and enablers to vaccine uptake is essential to implement sustainable, evidence-based strategies. This study aimed to evaluate the determinants of influenza vaccine acceptance and the impact of targeted interventions on improving uptake in a tertiary care hospital in India.

**Figure:**
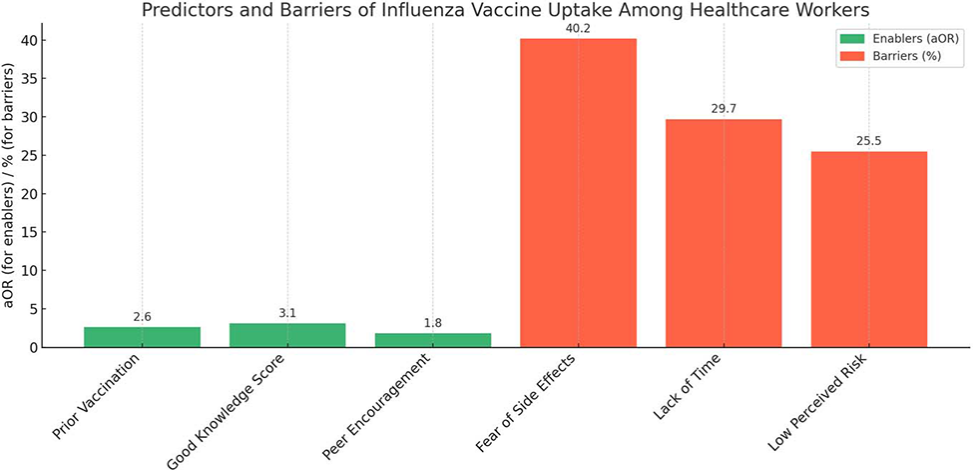
Predictors and Barriers of Influenza Vaccine Uptake Among Healthcare Workers This bar chart illustrates enablers (in green) and barriers (in red) influencing influenza vaccine uptake among healthcare workers at a tertiary hospital in South India. Key enablers identified through multivariate logistic regression include good knowledge (aOR: 3.1), prior vaccination (aOR: 2.6), and peer encouragement (aOR: 1.8). Major barriers included fear of side effects (40.2%), lack of time (29.7%), and low perceived risk (25.5%).

**Methods:**

A prospective, mixed-methods study was conducted between January and December 2023 at a 650-bed tertiary care teaching hospital in South India. A pre-tested questionnaire assessed knowledge, attitudes, and vaccination history of doctors, nurses, and paramedical staff. Interventions included mobile vaccine carts, departmental peer champions, behavioral nudges, and a mild reward-based incentive system. Pre- and post-intervention uptake data were collected and analyzed. Chi-square test was used for group comparisons, and multivariate logistic regression identified predictors of vaccine acceptance. A p-value < 0.05 was considered statistically significant.

**Results:**

Out of 734 HCWs surveyed, baseline influenza vaccination coverage was 30.5%. Following intervention, coverage rose to 70.2% (p < 0.001). Factors positively associated with vaccine uptake included prior vaccination (aOR: 2.6, 95% CI: 1.9–3.7), good knowledge score (aOR: 3.1, 95% CI: 2.2–4.5), and peer encouragement (aOR: 1.8, 95% CI: 1.2–2.7). Key barriers included fear of side effects (40.2%), lack of time (29.7%), and low perceived risk (25.5%).

**Conclusion:**

Multifaceted interventions significantly improved influenza vaccination among HCWs in a resource-limited tertiary hospital. Enhancing access, education, and peer influence, coupled with data-driven behavior modeling, offers a scalable model for improving vaccine uptake.

**Disclosures:**

All Authors: No reported disclosures

